# Neutralizing Antibodies Against the Porcine Endogenous Retroviruses (PERVs)

**DOI:** 10.3390/v17111437

**Published:** 2025-10-29

**Authors:** Jinzhao Ban, Ludwig Krabben, Benedikt B. Kaufer, Joachim Denner

**Affiliations:** Institute of Virology, Free University Berlin, 14163 Berlin, Germany; jinzhao.ban@fu-berlin.de (J.B.); ludwig.krabben@fu-berlin.de (L.K.); benedikt.kaufer@fu-berlin.de (B.B.K.)

**Keywords:** porcine endogenous retroviruses (PERVs), transmembrane envelope protein, surface envelope protein, neutralizing antibodies, epitope mapping, human immunodeficiency virus-1 (HIV-1)

## Abstract

Xenotransplantation using pig cells, tissues or organs may be associated with the transmission of porcine zoonotic or xenozoonotic microorganisms. Porcine endogenous retroviruses (PERVs) pose a special risk for xenotransplantation as these viruses can infect human cells and are integrated in multiple copies in the genome of all pigs and, therefore, they cannot be eliminated as other viruses can. To prevent PERV transmission to the recipient, several strategies have been developed: PERV-C-free animals, siRNA and genomic editing. Another strategy is the generation of vaccines based on neutralizing antibodies in order to protect the recipient. To investigate whether a protective vaccine is feasible in the case of PERV, the recombinant transmembrane (p15E) and the surface envelope (gp70) protein of PERV were cloned, produced, purified and used to immunize rats. For the first time, an adjuvant type that is approved for human use was used. In all cases we obtained virus binding antibodies as shown in Western blot assays and neutralizing antibodies as shown in neutralization assays, indicating the potential for a protective vaccine. The epitopes recognized by the antisera against p15E were determined using overlapping peptides. Two main epitopes were found in the sequence of p15E, one in the membrane proximal external region (MPER) and one in the fusion peptide proximal region (FPPR). The epitopes correspond to epitopes determined previously when immunizing different animal species with p15E of PERV. Antibodies against these epitopes block the conformational changes in the transmembrane envelope proteins that are required for membrane fusion, thereby inhibiting infection. The epitope in the MPER is related by sequence and location to an epitope in the transmembrane envelope protein of the human immunodeficiency virus-1 (HIV-1) recognized by a broadly neutralizing antibody from infected patients.

## 1. Introduction

Xenotransplantation, using genetically modified pigs, offers a promising solution to the shortage of allogeneic donor organs. Preclinical trials have demonstrated long survival times in non-human primates transplanted with hearts and kidneys from genetically modified pigs [1]. Moreover, the first transplantations of pig organs into human patients and brain-dead individuals indicate that this emerging technology is steadily progressing toward clinical application. Genetically modified pigs are needed to prevent hyperacute, antibody-mediated and cellular rejection as well as to preserve coagulation [2]. Furthermore, it is essential to prevent transmission of potentially zoonotic or xenozoonotic porcine microorganisms to the recipient [3,4]. The transmission of the porcine cytomegalovirus/porcine roseolovirus (PCMV/PRV) to the first human patient who received a pig heart in Baltimore, which contributed to the death of the patient [5], indicates the importance of preventing such transmissions. Whereas PCMV/PRV and many other porcine viruses can be eliminated by selection of virus-negative animals, early weaning, colostrum deprivation, treatment with antiviral drugs or vaccines if available, porcine endogenous retroviruses (PERVs) cannot. The proviruses are integrated with up to 60 copies in the genome of all pigs.

PERV-A and PERV-B are present in all pigs, whereas PERV-C is not found in all pigs [6,7]. While PERV-A and PERV-B have been shown to infect human cells under in vitro conditions, PERV-C is an ecotropic virus that infects only pig cells. Recombination between PERV-C and PERV-A results in recombinant viruses with a high replication rate and the capacity to infect human cells [8]. To date, there is no evidence of transmission of these viruses in either preclinical studies or first human xenotransplantations (for review see [9]). Despite this, various strategies have been developed to prevent PERV transmissions. First of all, the selection of PERV-C-negative animals to prevent PERV-A/C recombination. This strategy was used when PERV-C-free Auckland Island pigs were selected [10]. Auckland Island pigs represent an inbred population of feral pigs isolated on the sub-Antarctic island for over 100 years. The animals have been maintained under pathogen-free conditions in New Zealand; they are virologically well characterized and have been used as donor sources in first clinical trials of porcine neonatal islet cell transplantation for the treatment of human diabetes patients. In the first clinical trials, no porcine viruses, including PERVs were transmitted to the human recipients. Auckland Island pig kidney cells (selected to be free of PERV-C) were imported from New Zealand, and founder animals were established by somatic cell nuclear transfer (SCNT) at the Ludwig Maximilian University of Munich (LMU) [11].

Another strategy is based on RNA interference. PERV-specific small hairpin (sh) RNA directed against sequences in the polymerase gene (pol) were expressed in pig cells in vitro and in transgenic animals in vivo and reduced the PERV expression [12,13,14]. Although gene editing by zinc finger nucleases (ZFN) did not succeed [15], using clustered regularly interspaced short palindromic repeats/CRISPR-associated 9 (CRISPR/Cas9) resulted in the inactivation of all PERV proviruses in vitro [16] and in vivo [17].

Another promising strategy is the generation of vaccines against PERVs in order to protect the recipient. Vaccines against the murine leukemia virus (MuLV) and the feline leukemia virus (FeLV), two gammaretroviruses closely related to PERV, were able to prevent leukemia in mice [18] and cats [19], respectively. A total of 10 µg of the surface envelope protein gp70 of the Friend-MuLV coupled to keyhole limpet hemocyanin by glutaraldehyde protected more than 90% of the mice from erythroleukemia [18]. Live and inactivated vaccines against FeLV were found efficacious in reducing clinical signs in cats [19].

In previous immunization studies using recombinant p15E and gp70 of PERV, binding and neutralizing antibodies were induced in different species [20,21,22,23,24]. In those studies, however, Freund’s adjuvant was applied, which is not suitable for use in humans. To extend these findings, we immunized rats with newly produced and purified recombinant p15E and gp70 in combination with a type of adjuvant approved for human use. AddaVax (InvivoGen, Toulouse, France) was employed, a squalene-based oil-in-water nano-emulsion similar to MF59 that has been licensed for use in adjuvanted influenza vaccines [25]. In addition, a modified neutralization assay and a novel method for epitope mapping were applied.

## 2. Materials and Methods

### 2.1. Rats, Normal Rat Sera and Cells

Wistar rats were maintained and immunized by the Davids Biotechnologie GmbH (Regensburg, Germany). Five rats were immunized with the transmembrane envelope protein p15E, and five rats with the surface envelope protein gp70. Additional normal Wistar rat sera were obtained from the Institute for Animal Welfare, Animal Behavior and Laboratory Animal Science, Department of Veterinary Medicine, Free University Berlin.

Human embryonic kidneys 293T cells (from the Institute of Virology, Free University, Berlin) were cultured in Dulbecco’s modified eagle medium (DMEM) (Thermo Fisher Scientific, Waltham, MA, USA) supplemented with 10% heat-inactivated fetal bovine serum (FBS, PAN-Biotech, Aidenbach, Germany) Penicillin and streptomycin were not added to the culture medium.

### 2.2. Cloning, Expression and Purification of the Recombinant Proteins

**Figure 1:**
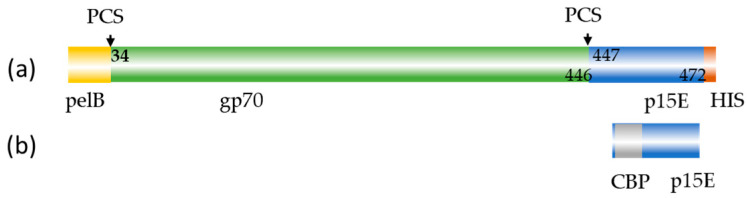
Schematic presentation of the recombinant envelope proteins used for immunization. (**a**) recombinant gp70 protein with pelB leader sequence, partial sequence of p15E and His-tag; and (**b**) recombinant p15E protein with calmodulin binding protein (CBP). PCS, protease cleavage site, numbering according to accession number AJ133817.

Two antigens were prepared and used for screening assays and immunization: the recombinant transmembrane envelope protein p15E and recombinant surface envelope protein the gp70 (Figure 1).

The ectodomain sequence of p15E (amino acids 447-477, accession numberHQ688786) was cloned into the pCal-n vector (Stratagene Europe, Amsterdam, The Netherlands) [20]. The recombinant plasmid was transferred to *Escherichia coli* DH5α (*E. coli* DH5α) competent cells. Single colonies were selected for sequencing, then the recombinant p15E was expressed in *E. coli* BL21 DE3 and induced with 0.2 mM isopropyl β-D-thiogalactopyranoside (IPTG) (Thermo Fisher Scientific, Waltham, MA, USA) in Luria–Bertani (LB) medium at OD_600_ of 0.6. After incubation for 3 h at 37 °C with shaking at 200 rpm, cells were harvested by centrifugation at 6000× *g* for 30 min, at 4 °C using a Sorvall RC 6 Plus Superspeed Centrifuge (Thermo Fisher Scientific, Waltham, MA, USA). Cells were lysed by incubation in 50 mM Tris/HCl (pH 7.5), 150 mM NaCl, 2 mM CaCl_2_ and 10 mM dithiothreitol (DTT), supplemented with 1 mg/mL lysozyme, 1000 U benzonase (Merck, Darmstadt, Germany) and cOmplete ethylenediaminetetraacetic acid (EDTA)-free protease inhibitor cocktail (Merck, Darmstadt, Germany) for 30 min at room temperature. Subsequently, the lysate was subjected to 20 s sonification cycles with 1 min cooling on ice. The suspension was diluted in a 1:1 ratio with binding buffer (50 mM Tris/HCl (pH 7.5), 150 mM NaCl, 2 mM CaCl_2_) and centrifuged at 50,000× *g* for 20 min; supernatant was filtered through a 0.45 µm filter. The protein was purified by calmodulin resin affinity chromatography using an ÄKTAprime plus system (both from Cytiva Life Sciences, Freiburg, Germany) with 5 mM DTT included in binding buffer and 2 mM DTT in the washing (50 mM Tris/HCl, pH 7.5, 150 mM NaCl, 2 mM CaCl_2_) and elution buffers (50 mM Tris/HCl, pH 7.5, 150 mM NaCl, 2 mM EGTA).

The entire sequence of the surface envelope protein gp70 of PERV-A (amino acids 34-446, accession number Q688785) and a short stretch of the ectodomain sequence of p15E were cloned into pET22b (+) vector (Merck, Darmstadt, Germany) [21]. This part of the p15E sequence was included to make the antigen similar to the sequence of the recombinant gp70/p15E of FeLV successfully used as Leucogen to vaccinate cats [26]. The pET22b (+)-gp70 was transferred into *E. coli* DH5α competent cells. Confirmative sequencing was performed after single-colony selection. The recombinant protein was expressed in *E. coli* BL21 DE3 cells and induced with 0.1 mM IPTG in LB medium at an OD_600_ of 0.6. After incubation for 3 h at 37 °C with shaking at 200 rpm, cells were harvested as mentioned above. *E. coli* DH5α and BL21 strains were grown on LB agar or in LB medium with ampicillin (100 µg/mL) as the selective antibiotic [21]. Cells were lysed in phosphate-buffered saline (PBS) (pH 7.4) with 10 mM DTT, supplemented with 1 mg/mL lysozyme by 20 s sonification cycles with 1 min intervals on ice. Cells were harvested by centrifugation at 22,000× *g* for 30 min at 4 °C and the insoluble pellet was lysed by incubation in lysis buffer (pH 8.0) (100 mM NaH_2_PO4, 10 mM Tris/HCl 6 M GuHCl and 10 mM DTT, supplemented with 3 U/µL benzonase) overnight at 4 °C. The supernatant was filtered through a 0.45 µM filter. Purification was performed by Ni-NTA resin affinity chromatography (Cytiva Life Sciences, Freiburg, Germany) using an ÄKTAprime plus system with 5 mM DTT included in binding (pH 8.0), two washing (pH 6.3 and pH 5.0) and elution buffers (pH 4.2) (100 mM NaH_2_PO_4_, 10 mM Tris/HCl, 8 M urea). Purified recombinant gp70 was concentrated to 1 mg/mL using Vivaspin 15R Centrifugal Concentrator (Vivaproducts, Littleton, CO, USA) and subsequently dialyzed against PBS using dialysis cassettes (15 mL, Thermo Fisher Scientific, Waltham, MA, USA). Recombinant proteins were characterized by sodium dodecyl sulfate-polyacrylamide gel electrophoresis (SDS-PAGE) and Western blotting.

### 2.3. Immunization Schedule

Wistar rats (*n* = 5 per group) were immunized with 300 µg of recombinant p15E or gp70 emulsified in AddaVax (InvivoGen, Toulouse, France) for the primary immunization, followed by two booster injections of the same dose emulsified in the same adjuvant on days 14 and 28. Preimmune sera were collected one day before immunization and immune sera were collected on day 42 after the first immunization. The animal immunizations were conducted by Davids Biotechnologie GmbH (Regensburg, Germany).

### 2.4. SDS-PAGE

Protein expressions and purifications were analyzed by SDS-PAGE, which was performed using a 17% separating gel for p15E and a 12% separating gel for gp70. A total of 1 µg p15E and 100 ng gp70 were mixed with 6 × SDS-PAGE loading buffer and denatured at 95 °C for 10 min in a metal heating block (Eppendorf Vertrieb Deutschland GmbH, Wesseling-Berzdorf, Germany). Proteins were separated by SDS-PAGE and stained by Imperial Protein Stain (Thermo Fisher Scientific, Waltham, MA, USA) for 1 h at room temperature and destained with double-distilled water (ddH_2_O) until background was clear.

### 2.5. Western Blotting

SDS-PAGE was performed as described above. The proteins were transferred onto 0.2 µm PVDF membranes (Carl Roth GmbH, Karlsruhe, Germany) using a semi-dry transfer system (Peqlab Biotechnologie GmbH, Erlangen, Germany) at a constant current of 0.13 A for 70 min. A pre-stained protein ladder, 10 to 250 kDa (Thermo Fisher Scientific, Waltham, MA, USA) was used. The blot was blocked with 5% (*w*/*v*) non-fat dry milk (NFDM) powder in Tris-buffered saline with 0.05% Tween-20 (TBST) for 1 h at room temperature. Membranes were then incubated with positive control sera (goat sera anti-p15E, #355 and anti-gp70, #62 [21]), rat anti-p15E (1:1000) or rat anti-gp70 (1:1000) overnight at 4 °C, followed by incubation with the corresponding horseradish peroxidase (HRP)-conjugated donkey anti-goat IgG antibody (1:20,000) (Merck, Darmstadt, Germany) and HRP-conjugated goat anti-rat IgG (H + L) secondary antibody (1:10,000) (Thermo Fisher Scientific, Waltham, MA, USA) for 1 h at room temperature. Membranes were washed three times for 10 min with TBST between each incubation step. Blots were developed with ECL Prime Western Blot Detection Reagent (Cytiva Life Sciences, Freiburg, Germany) according to the manufacturer’s instructions.

### 2.6. In Vitro Neutralization Assays

293T cells cultured in DMEM supplemented with heat inactivated 10% FBS at 37 °C in a 5% CO_2_ incubator were used for neutralization assays. Rat anti-p15E and anti-gp70 sera were heat-inactivated at 56 °C for 30 min and serially diluted two-fold in DMEM. A total of 20 µL undiluted or serially diluted sera and 80 µL PERV supernatant (threshold cycle (Ct) < 25) were mixed and incubated at 37 °C for 15 min. The mixture was added to 100 µL HEK 293T cells (5000 cells/100 µL) in 96-well plates and incubated for 96 h. Lysis duplex real-time polymerase chain (real-time PCR) reaction detecting both, PERV-pol and human glyceraldehyde-3-phosphate dehydrogenase (GAPDH) was performed in situ. Direct PCR Lysis Reagent (Cell) (Viagen Biotech, Los Angeles, CA, USA) and Nuclease-free water were mixed at a 1:1 ratio and Proteinase K (Viagen Biotech, Los Angeles, CA, USA) was added at 800µg per 1 mL of the resulting mixture. This mixture was applied to lyse cells in situ, adding 60 µL per well. The cell lysates were incubated overnight at 55 °C for lysis and wrapped in damp paper towels to prevent evaporation, followed by heat inactivation of proteinase K at 85 °C for 1.5 h. The resulting products were used directly for real-time PCR analysis. Differences among sera were assessed using one-way ANOVA followed by Dunnett’s multiple comparisons test comparing immune rat sera with the normal rat serum. Statistical significance was defined as *p* < 0.05.

### 2.7. Duplex Real-Time PCR for the Detection of Provirus

To determine the provirus in 293T cells, a duplex real-time PCR was performed. Genomic DNA was extracted using the method described above. Real-time PCR reactions were carried out using SensiFAST Probe No-ROX kit (Meridian Bioscience, Cincinnati, OH, USA), specific primers and probes (Table 1) on a qTOWER^3^G qPCR cycler (Analytik Jena, Jena, Germany). The cycling conditions used were as follows: initial denaturation at 95 °C for 5 min, followed by 45 amplification cycles of denaturation at 95 °C for 15 s, annealing at 62 °C for 30 s and extension at 72 °C for 30 s.

### 2.8. Epitope Mapping

The ectodomain of PERV p15E (EPISLTLAVMLGLGVAAGVGTGTAALITGPQQLEKGLGEHAAMTEDLRLEESVSNLEESLTSLSEVVLQNRRGLDLLFLREGLCAALKEECCFYVDHSGAIRDSMSKLRERLERRRRREADQGWFENRSPWMTTLLSALTGPLVVLLLLLT) was synthesized as 74 peptides of 13-mers overlapping by 11 amino acids (JPT Peptide Technologies, Berlin, Germany) (Supplementary Table S1). N-terminal acetylation of the peptides makes the peptides more stable against N-terminal degradation, and the uncharged N-acetyl group corresponds more closely to the structure of the native antigen than a charged NH_3_^+^ group.

Peptides were covalently immobilized on the glass surface. An optimized hydrophilic linker moiety is inserted between the glass surface and the antigen derived peptide sequence to avoid false negatives caused by steric hindrances. For technical reasons all peptides contain a C-terminal glycine.

The profiling experiment was performed with a total of five rat serum samples diluted to 1:40, 1:200 and 1:1000 with blocking buffer and incubated for 1 h at 30 °C on the Multiwell microarray slide. Each slide contained 21 individual mini-arrays (1 mini-array per sample dilution).

Subsequent to sample incubation, a secondary, fluorescently labeled anti-rat-IgG antibody, at 0.1 μg/mL, was added into the corresponding wells and left to react for 1 h.

Additional control incubations applying the secondary antibody only (but no rat serum sample) were performed in parallel on the same microarray slide to assess false positive binding of the secondary antibody to peptides.

Serum samples were diluted in blocking buffer (Pierce International, Superblock TBS T20) and applied to microarrays for 1 h at 30 °C, then they were incubated with secondary antibody diluted in blocking buffer for 1 h at 30 °C. Secondary antibody used for detection: Anti-rat IgG (Jackson Immunoresearch, Ely, United Kingdom), label Cy5, applied concentration 0.1 μg/mL. Before each incubation step, microarrays were washed with washing buffer (50 mM TBS-buffer including 0.1% Tween-20, pH 7.2). Finally, the microarrays were dried.

After washing and drying, the microarrays were scanned using a high-resolution fluorescence scanner (Axon Genepix Scanner 4300 SL50, Molecular Devices, San Jose, CA, USA) at 635 nm to obtain fluorescence intensity profiles. Laser settings and applied resolution were identical for all measurements. The resulting images were analyzed und quantified using spot-recognition software GenePix Pro 7 (Molecular Devices). For each spot, the mean signal intensity was extracted (between 0 and 65,535 arbitrary units).

For further data evaluation, the so called MMC2 values were determined. The MMC2 equals the mean value of all three instances on the microarray except when the coefficient of variation (CV)—standard-deviation divided by the mean value—is larger 0.5. In this case the mean of the two closest values (MC2) is assigned to MMC2.

## 3. Results

### 3.1. Production and Purification of the Antigens

**Figure 2:**
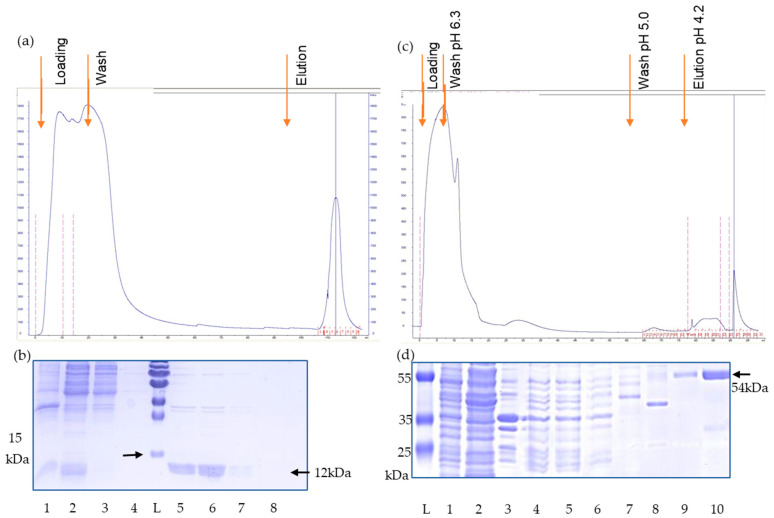
(**a**) Elution profile of the calmodulin binding protein (CBP) affinity chromatography of recombinant p15E. (**b**) Coomassie blue staining of SDS-PAGE of fractions of the elution process L, protein ladder, 1, lysed cells, 2, supernatant, 3, flow through, 4, wash fraction, 5–8 elution fractions, i.e., recombinant p15E (12 kDa) protein purified by affinity chromatography. (**c**) Elution profile of the His tag affinity chromatography of recombinant gp70. (**d**) Coomassie blue staining of the SDS-PAGE of fractions of the elution process. L, protein ladder, 1, lysed cells, 2, soluble fraction, 3, insoluble fraction, 4, 5, cleared lysate filtered by 0.45µM filter, 6, flow through, 7, wash fraction, pH 6.3, 8, wash fraction at pH 5.0, 9, 10, elution fractions at pH 4.2, i.e., recombinant gp70 (54 kDa) purified by affinity chromatography.

To investigate whether a protective vaccine based on neutralizing antibodies against the envelope proteins is feasible in the case of PERV, recombinant envelope proteins were produced. To prepare the antigens required for immunization and testing, the DNA sequence of p15E or gp70 were cloned into vectors pCal-n or pET22b (+), respectively (Figure 1), then protein was expressed in *E. coli* and subsequently purified using calmodulin resin or Ni-NTA resin affinity chromatography under denatured conditions. Elution fractions showed a strongly enriched band at approximately 12 kDa and a single band at 54 kDa corresponding to the expected size of the recombinant p15E and gp70, respectively (Figure 2). This indicates that the recombinant proteins were successfully expressed and purified.

### 3.2. Characterization of the Antigens

**Figure 3:**
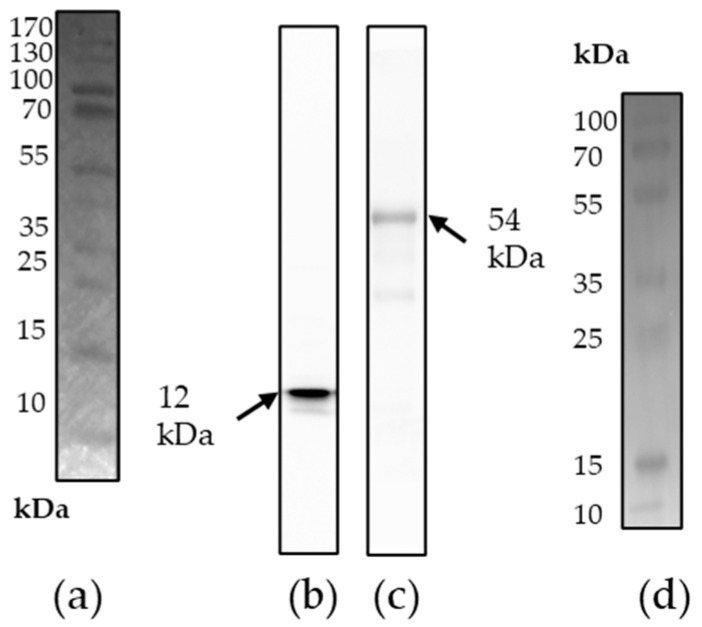
(**a**) A pre-stained protein ladder was used to indicate the molecular weight of the recombinant p15E. (**b**) Western blot analysis of recombinant p15E (12 kDa) protein. Goat anti-p15E serum (1:1000) was used as primary antibody, donkey anti-goat IgG antibody (1:20,000) as secondary antibody. (**c**) Western blot analysis of recombinant gp70 (54 kDa) protein. Goat anti-gp70 serum (1:1000) was used as primary antibody, donkey anti-goat IgG antibody (1:20,000) as secondary antibody. (**d**) A pre-stained protein ladder was used to indicate the molecular weight of the recombinant gp70.

To verify the identity of the purified recombinant p15E and gp70, a Western blot was performed using goat anti-p15E (goat serum #355 [21]) and goat anti-gp70 (goat serum #62 [21]) antibodies. The blot showed single bands at 12 kDa and 54 kDa, corresponding to the expected molecular weights of recombinant p15E and gp70, respectively. These results confirm that the recombinant proteins were successfully expressed and purified without significant degradation (Figure 3).

### 3.3. Detection of Binding Antibodies

**Figure 4:**
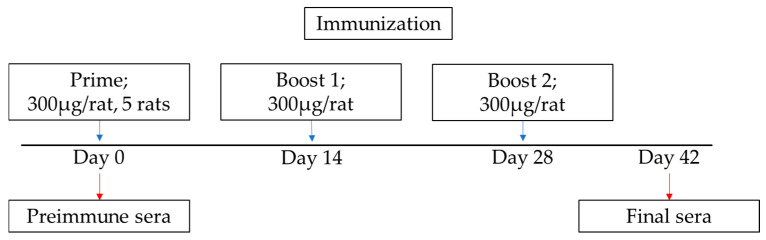
Scheme of immunization and sample collection. Wistar rats were immunized with 300 µg purified p15E or gp70 protein mixed with the adjuvant AddaVax per rat for the primary immunization and followed by two boosts. Pre-immune sera were collected before the immunization. On day 42 final sera were collected.

Five rats were immunized with the recombinant protein p15E, another five rats were immunized with the recombinant protein gp70 according to the immunization schedule (Figure 4).

**Figure 5:**
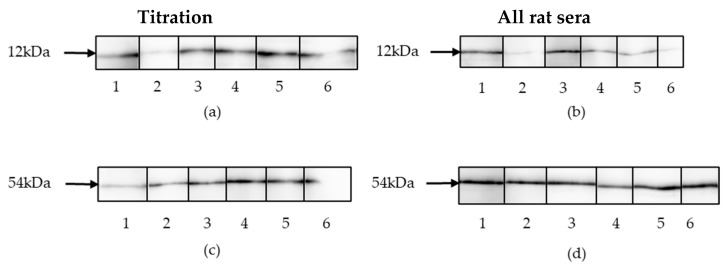
Results of the Western blot analysis. (**a**) Titration of rat serum 1 against p15E using purified recombinant p15E protein as antigen. 1, goat serum against p15E, followed by dilutions of the rat serum 2, 1:100; 3, 1:200; 4, 1:500; 6, 1:1000; 6, 1:10,000. (**b**) Western blot analysis of 5 rat sera anti-p15E using purified recombinant p15E protein as antigen. 1, goat serum against recombinant p15E; 2, rat serum 1; 3, rat serum 2; 4, rat serum 3; 5, rat serum 4; 6, rat serum 5; all at dilution 1:1000. (**c**) Titration of rat serum 1 against gp70 using purified recombinant gp70 protein as antigen. 1, goat sera anti-gp70; followed by dilutions of the rat sera 2, 1:100; 3, 1:200; 4, 1:500; 5, 1:1000; 6, 1:10,000. (**d**) Western blot analysis of rat sera anti-gp70 using purified recombinant gp70 protein as antigen. 1, goat sera against recombinant gp70; 2, rat serum 1; 3, rat serum 2; 4, rat serum 3; 5, rat serum 4; 6, rat serum 5, all at dilution 1:1000.

To analyze whether specific antibodies against the recombinant proteins p15E or gp70 are present in the rat sera, Western blots were performed using control goat sera. The control sera were obtained previously by immunization of goats with recombinant p15E or gp70 [21]. In addition, to determine the titer of the specific antibodies in rat sera against antigens in the Western blot assay, rat sera were diluted at 1:100, 1:200, 1:500, 1:1000 and 1:10,000. The Western blot analysis showed single bands at 12kDa or 54kDa, which are the theoretical molecular weights of recombinant p15E or gp70. As expected, all rats produced specific antibodies against the recombinant protein p15E or gp70, and the titer of the rat sera against recombinant p15E or gp70 was at least 1:10,000 (Figure 5). This antibody titer is comparable with the titers found in previous immunizations [20,21].

### 3.4. Detection of Neutralizing Antibodies

**Figure 6:**
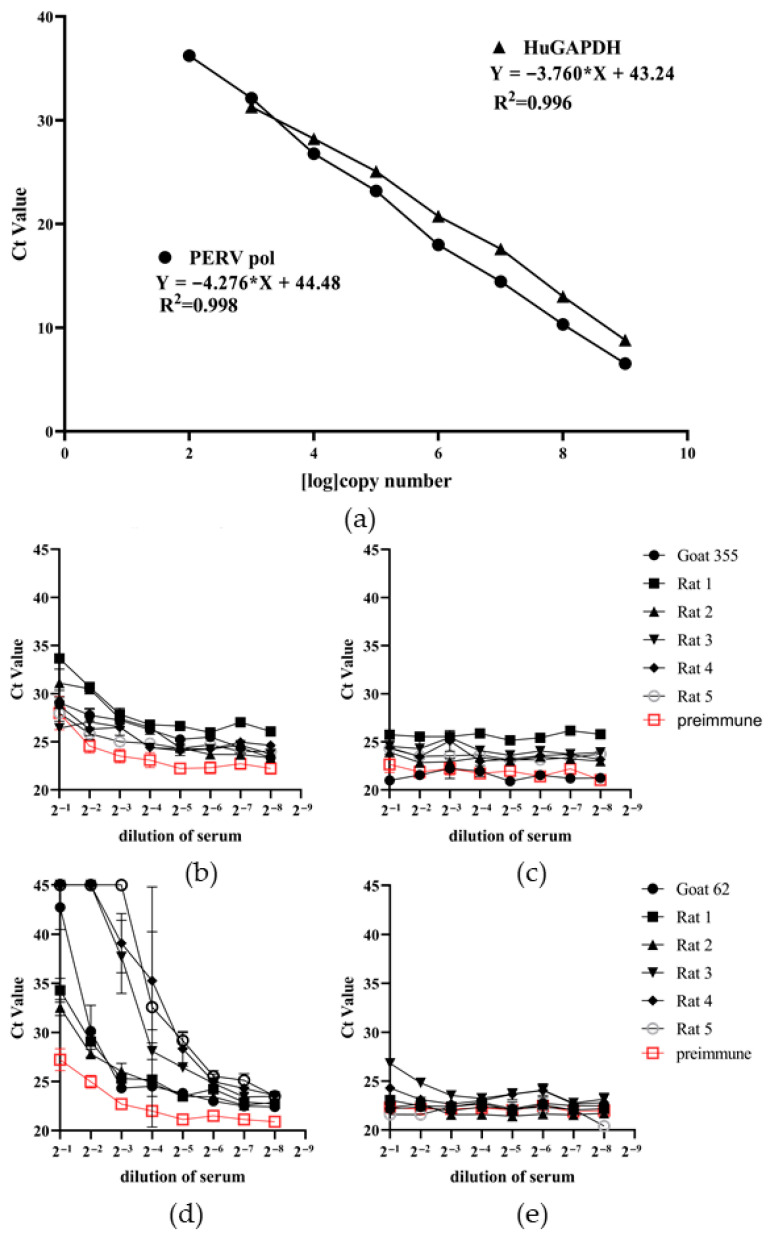
Results of the neutralization assays. (**a**) Efficacy of the duplex real-time PCR used in the neutralization assay. A synthetic Gene block containing the sequences of the PERV pol region and of huGAPDH was used in 10-fold dilution and a duplex real-time PCR was performed using primers and probes for PERV pol and huGAPDH. (**b**) The neutralization capacity of five rat sera against p15E, a preimmune serum and a control goat serum against p15E (serum #355) was measured detecting PERV pol using a duplex real-time PCR. (**c**) Corresponding ct values of huGAPDH. (**d**) The neutralization capacity of five rat sera against gp70, a preimmune serum and goat anti-gp70 (serum #62) were measured detecting PERV pol by duplex real-time PCR. (**e**) Corresponding ct values of huGAPDH. Concerning PERV neutralization: The higher the Ct value, the fewer viral genomes were detected, indicating that the antiserum more effectively inhibited viral infection. Data of the immune sera were analyzed by one-way ANOVA followed by Dunnett’s multiple comparisons test vs. the normal rat serum, all sera had a value below *p* < 0.05 and were considered significant. Concerning human GAPDH: Identical Ct values across all wells indicate that each well contained the same number of cells and that the antiserum was not cytotoxic.

To detect neutralizing antibodies against PERV in the immune rat sera, a neutralization assay was established. This assay is based on PERV infection in human 293T cells measuring provirus integration into cellular DNA. For this, a PCR method called lysis duplex real-time PCR was used. Cells were lysed directly in the 96-well plate. Supernatant from 293T cells producing a PERV-A/C virus which was adapted to human cells by passaging on 293T cells [28] was pre-incubated with immune serum dilutions and added to uninfected 293T cells. The real-time PCR used human GAPDH for evaluation. Since GAPDH is present in all cells this value indicates the total amount of cells, whereas the value for PERVpol indicated the number of infected cells. Sera from five rats immunized with recombinant p15E and sera from five rats immunized with the recombinant gp70 showed neutralizing capacity (Figure 6). The higher the ct value of PERVpol, the lower the number of infected 293 cells, indicating a greater neutralizing capacity (Figure 6b,d). The neutralization capacity was dose-dependent. Identical ct values for GAPDH confirmed that the number of 293 cells was consistent across all tested wells and that the antiserum was not cytotoxic (Figure 6c,e). Cytotoxicity may cause misleading results, appearing as false-positive neutralizing activity. The neutralizing activity of the antisera against p15E was much weaker compared to the neutralizing activity of the antisera against gp70 (Figure 6).

### 3.5. Results of the Epitope Mapping

To identify the epitopes in PERV p15E recognized by the immune rat sera, an epitope mapping was performed using 74 overlapping 13-mer peptides (with 11 amino acids overlaps) spanning the ectodomain of p15E. The peptides immobilized on microarray slides were incubated with rat sera and with a secondary, fluorescently labeled anti-rat-IgG antibody. The microarrays were scanned using a high-resolution fluorescence scanner (Axon Genepix Scanner 4300 SL50, Molecular Devices, San Jose, CA, USA). Laser settings and applied resolution were identical for all performed measurements. The resulting images were analyzed and quantified using spot-recognition software. For each spot, the mean signal intensity was extracted (between 0 and 65,535 arbitrary units) (Supplementary Table S1).

**Figure 7:**
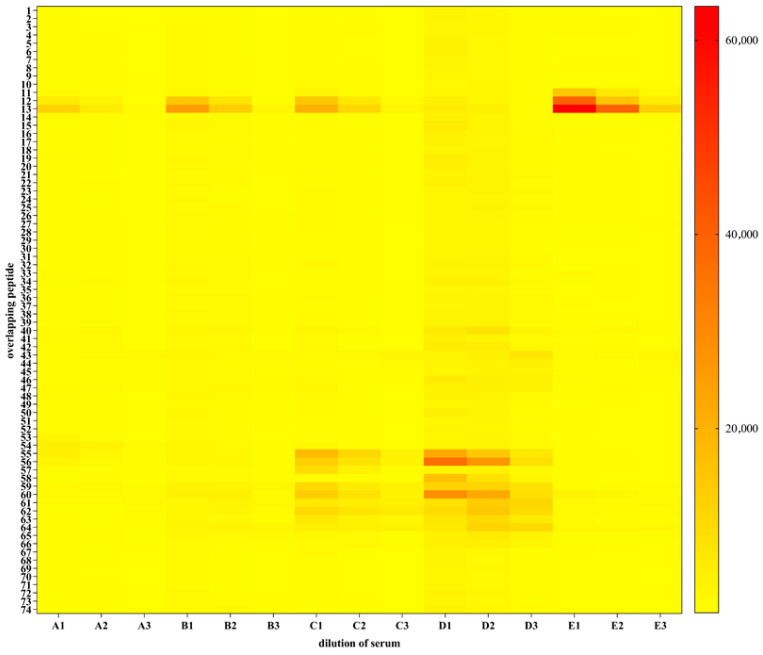
Epitope mapping of five rat sera against p15E. Heatmap diagram showing all sample incubations; y-axis represents peptide sequences in the library (see Supplementary Table S1), x-axis specifies samples applied. The MMC2 values are shown color coded ranging from white (0 or low intensity) via yellow (middle intensity) to red (high intensity). A, rat serum 1; B, rat serum 2; C, rat serum 3; D, rat serum 4; E, rat serum 5. 1, serum dilution 1:40; 2, serum dilution 1:200; 3, serum dilution 1:1000. Strong signals were obtained with control spots containing the full-length rat IgG demonstrating a correct assay performance.

To visualize the results obtained and to compare binding regions across the individual incubations, a heatmap diagram (Figure 7) was computed showing fluorescence intensities in a color-coded manner from white (no binding) to red (strong binding).

**Figure 8:**
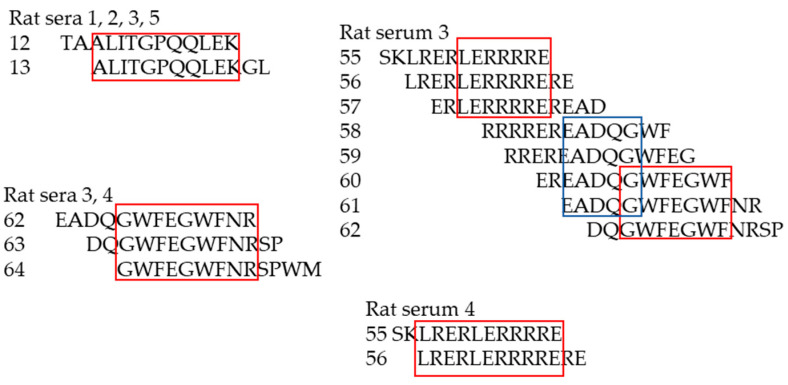
Determination of the epitopes recognized by rat sera 1, 2, 3, 4 and 5. The numbers of the rats and of the peptides are shown and the epitopes are framed. The red boxes indicate major epitopes, the blue box a potential minor epitope.

Three of the five investigated rat sera (namely rat serum, 1, 2 and 5) showed a comparable binding profile indicating one putative epitope represented within peptide 12 and 13 (peptide 12 TAALITGPQQLEK, peptide 13 ALITGPQQLEKGL), indicating epitope ALITGPQQLEK (Figure 8). In addition to the peptides 11, 12 and 13, the incubation of rat serum 3 yielded significant signals in a region spanning peptides 55 to 63. Probably more than one antibody from the serum is responsible for the detected signal distribution (Figure 8). Rat serum 4 gave rise to several signals with intensities above 5000 more or less all over the peptide scan. The most prominent region with the strongest signals (above 20,000) is represented by peptide 55 and 56 (Figure 8). Therefore, three main epitopes were detected, ALITGPQQLEK, in the FPPR, as well as LERRRRE/LRERLERRRRERE and GWFEGWF/GWFEGWFNR in the MPER of p15E.

**Figure 9:**
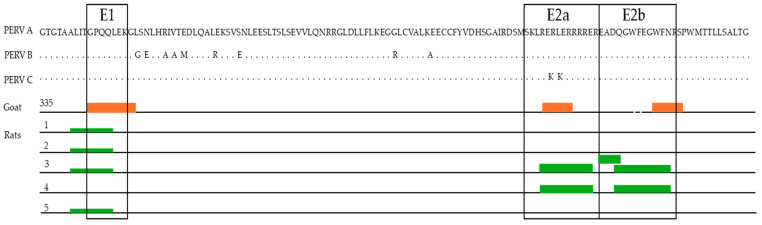
Summary of epitopes recognized by five rat sera (green) and one control goat serum (orange, goat #355) against PERV p15E. The epitopes recognized by goat serum 355 were determined previously using another method, based on a cellulose-adsorbed peptide spot library of 15-mer peptides overlapping by 12 amino acids and detection by chemiluminescence [21].

For the first time, we demonstrate that immunization with the AddaVax adjuvant also induces neutralizing antibodies. Notably, sera against p15E recognized epitopes similar to those previously reported in studies using CFA/IFA (Figure 9). Previously, epitope mapping was carried out using a cellulose-adsorbed peptide spot library consisting of 15-mer peptides overlapping by 12 amino acids, with detection performed by chemiluminescence [20,21]. In the present study, a library of 74 peptides composed of 13-mers overlapping by 11 amino acids was employed. Shorter peptides are expected to allow more precise fine mapping of the epitopes. All peptides were covalently immobilized on a glass surface, and epitope detection was performed using a fluorescently labeled anti-rat IgG antibody in combination with a high-resolution fluorescence scanner (Axon Genepix Scanner 4300 SL50, Molecular Devices, San Jose, CA, USA). The epitopes identified by both approaches were highly similar (Figure 9).

## 4. Discussion

Immunization of rats with recombinant PERV p15E and PERV gp70 induced neutralizing antibodies, indicating that these envelope proteins may serve as effective antigens in vaccines designed to protect against PERV infection.

These findings are consistent with previous studies in which goats, rats, mice and guinea pigs were immunized with both proteins, despite several differences between those studies and the present immunization approach.

**Figure 10:**
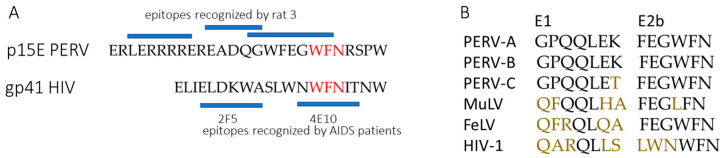
(**A**) Sequence comparison between the membrane proximal external region (MPER) of the transmembrane envelope protein PERV p15E and HIV-1 gp41. Identical amino acids are marked in red. The epitopes recognized by the immune serum from rat 3 and the epitopes recognized by the monoclonal antibodies 2F5 and 4E10, isolated from AIDS patients [29] are shown as blue bars. (**B**) Sequence comparison of epitopes E1 and 2Eb of different retroviruses. Non-conserved amino acids are shown in color.

Previously, goats were immunized with 500 µg antigen, Wistar rats with 150 µg, Balb/c mice with 50 µg and Guinea pigs with 200 µg antigen [20,21]. In this study, we increased the antigen dose to 300 µg in order to achieve improved immunization outcomes in Wistar rats. The immunization schedule was also different. In previous studies a second and third immunization was performed after two and five weeks, here booster immunizations were performed after 14 and 28 days (Figure 4). The main difference is the usage of a different adjuvant. In previous studies complete Freund’s adjuvant (CFA) was used for immunization and incomplete Freund’s adjuvant (IFA) for booster immunizations [20,21]. CFA is composed of mineral oil (paraffin oil and mannide monooleate) and inactivated and dried mycobacteria (*M. tuberculosis*). CFA contains trehalose 6,6’ dimycolate, which stimulates the macrophage inducible Ca^2+^-dependent lectin receptor (Mincle). Additionally, CFA has ligands for toll-like receptor 2 (TLR2), TLR4 and TLR9. IFA lacks the mycobacterial components. CFA cannot be used in humans because it causes severe side effects, it triggers strong and long-lasting inflammatory reactions, and can lead to abscesses, ulcerations, necrosis and chronic granulomas at the injection site. Therefore, we tested AddaVax, which is a squalene-based oil-in-water nano-emulsion. Its formulation is similar to that of MF59 that has been licensed in Europe for adjuvanted flu vaccines [25]. Squalene is an oil more readily metabolized than the paraffin oil used in Freund’s adjuvants. This class of adjuvants is believed to act through recruitment and activation of antigen-presenting cells (APCs) and stimulation of cytokines and chemokines production by macrophages and granulocytes [25]. As shown in Figure 9, immunization with AddaVax induced antibodies that recognized the same epitopes as those obtained previously using Freund’s adjuvant. It is of great interest that the main epitope in the MPER of p15E shows a sequence homology with an epitope in gp41 of HIV-1, which is recognized by a broadly neutralizing monoclonal antibody, 4E10, isolated from an AIDS patient [29] (Figure 10). 4E10 neutralizes diverse subtypes of HIV-1 (e.g., subtypes B, C, and E) [29]. Not surprisingly, similar epitopes were also found when cats were immunized with p15E of FeLV [30].

**Figure 11:**
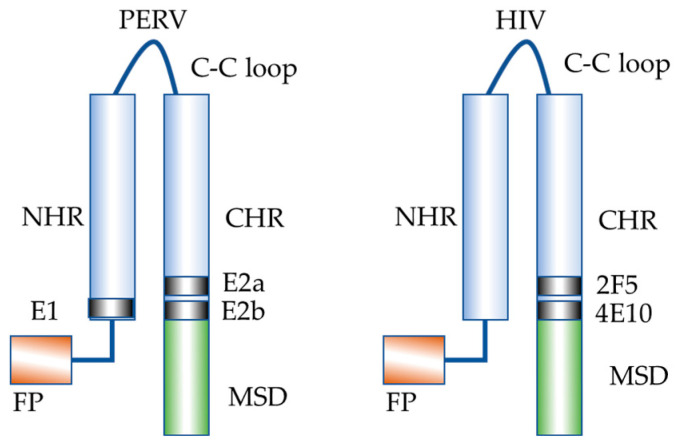
Localization of the epitopes in the transmembrane envelope proteins p15E and gp41 of PERV and HIV-1, respectively, after interaction of the CHR and NHR during infection. FP, fusion peptide; NHR, N-terminal helical region; C-C loop, cysteine-cysteine loop; CHR, C-terminal helical region; MSD, membrane spanning domain.

It is not only the partial sequence homology between the epitopes in the gamma retroviruses, PERV and FeLV on the one hand and HIV-1 on the other hand, but also the localization of these epitopes in the MPER (Figure 11), what is important for the understanding of the function of this domain. Antibodies directed against epitopes such as E1 and E2 in p15E in the case of PERV, and antibodies such as 2F5 and 4E10 that target similar epitopes in the MPER of gp41 of HIV-1, block the conformational changes in the corresponding transmembrane envelope proteins p15E and gp41 that are required for membrane fusion. As a result, fusion between the virus and the host cell is prevented, thereby inhibiting infection. In more recent studies with HIV-1 it has been demonstrated that the MPER is important for env-mediated fusion and virus infectivity [31,32]: mutations to Ala of three of five conserved Trp residues in this region are sufficient to abrogate syncytium formation [32]. In contrast to our success in inducing antibodies against the MPER of PERV (and FeLV), numerous attempts to induce antibodies of the type 2F5 and 4E10, broadly neutralizing HIV-1, failed [33,34,35]. Immunization experiments using the backbone of p15E of PERV in which the epitopes in the FPPR and MPER were substituted by the corresponding epitopes of 2F5 and 4E10 induced binding antibodies against the HIV epitopes, but despite the exact recognition of the 2F5 epitope, no or very weak neutralization of HIV-1NL4-3 by the immune sera was demonstrated [36].

In immunization studies using subunits of p15E, i.e., recombinant proteins corresponding to the N-terminal, the C-terminal helical region (NHR, CHR) and a p15E with a mutation in the Cys–Cys loop, no MPER-specific neutralizing antibodies were induced, indicating that the Cys-Cys loop is important [24]. However, when the animals were immunized with the FPPR/NHR subunit or the mutated p15E alone, novel neutralizing antibodies binding to the NHR were found. The epitope was IVTEDLQALEKS and was localized in the NHR at a position where in gp41 of HIV-1 the neutralizing antibodies D5 and HK20 are binding [37,38,39]. Such antibodies were not detected in this immunization study.

Since no animal models are available to study the efficacy of PERV-neutralizing antibodies in vivo, we immunized cats with the corresponding p15E protein of FeLV. This approach induced neutralizing antibodies that bound to epitopes similar to those recognized in PERV p15E [30]. Upon challenge with infectious FeLV, the immunized cats were protected from leukemia [40], demonstrating that these antibodies are capable of neutralizing the virus in vivo.

The neutralizing activity of antisera directed against gp70 of PERV was considerably stronger than that of antisera against p15E of PERV (Figure 6). Nevertheless, both antigens should be used for immunization, as previous studies have shown that combined immunization with gp70 and p15E induces significantly higher titers of antibodies neutralizing PERV than immunization with either antigen alone [22]. In addition, simultaneous immunization of rats with gp70 and p15E of FeLV resulted in higher titers of antibodies neutralizing FeLV compared with immunization using gp70 or p15E individually [41]. Moreover, since vaccination of cats with the transmembrane envelope protein of FeLV alone provided in vivo protection against infection [40], it can be assumed that immunization with both proteins will confer an even stronger protective effect.

Because immunization of different animal species with PERV p15E and gp70 consistently elicited neutralizing antibodies targeting identical epitopes critical for the infection process, it is reasonable to suggest that similar protective antibodies could also be induced in non-human primates and humans.

## 5. Conclusions

Immunization of rats with the recombinant transmembrane (p15E) and surface (gp70) envelope proteins of PERV induced both binding and neutralizing antibodies. The induction of such antibodies across multiple animal species as shown here and previously suggests that immunization of non-human primates and humans may similarly elicit protective responses. In the next step, non-human primates (rhesus monkeys or baboons) should be immunized to determine whether they also produce such neutralizing antibodies, prior to immunizing a baboon that will subsequently receive a pig heart transplant. Immunization was carried out using an adjuvant comparable to MF59, which is licensed for human use. The neutralizing antibodies targeted epitopes within the FPPR and MPER, regions that play a critical role in the infection process. This study is also relevant for the development of vaccines against HIV-1, as the epitope in the MPER shares sequence and positional similarity with an epitope in the transmembrane envelope protein of HIV-1 that is recognized by a broadly neutralizing antibody from infected patients.

## Figures and Tables

**Table 1:** Primers and probes used for the duplex real-time PCR.

Primer/Probe	Sequence 5′-3′	Direction	Location (Nucleotide Number)	Accession Number	Reference
PERV pol for	CGACTGCCCCAAGGGTTCAA	+	3568–3587	GenBank HM159246	Yang et al. [16]
PERV pol rev	TCTCTCCTGCAAATCTGGGCC	−	3783–3803		
PERV pol probe	6FAM-CACGTACTGGAGGAGGGTCACCTG-BHQ1	+	3655–3678		
GAPDH for	GGCGATGCTGGCGCTGAGTAC	+	365–385	GenBank AF261085	Behrendt et al. [27]
GAPDH rev	TGGTTCACACCCATGACGA	−	495–513		
GAPDH probe	HEX-CTTCACCACCATGGAGAAGGCTGGG-BHQ1	+	407–430		

for, forward primer; rev, reverse primer.

## Data Availability

Data is contained within the article or supplementary material.

## Supplementary Materials

The following supporting information can be downloaded at: https://www.mdpi.com/article/10.3390/v17111437/s1, Table S1: Overview of the overlapping peptides used for the epitope mapping and results of the binding experiments of five rat sera immunized with recombinant p15E at three different dilutions.

## Abbreviations

The following abbreviations are used in this manuscript:

AIDS	Acquired immunodeficiency syndrome
CHR	C-terminal helical region
CRISPR/Cas9	Clustered regularly interspaced short palindromic repeats/CRISPR-associated 9
DMEM	Dulbecco’s modified eagle medium
FeLV	Feline leukemia virus
FPPR	Fusion peptide proximal region
LMU	Ludwig Maximilian University of Munich
MPER	Membrane proximal external region
MuLV	Murine leukemia virus
NHR	N-terminal helical region
PERV	Porcine endogenous retrovirus
PCMV/PRV	Porcine cytomegalovirus/porcine roseolovirus
SCNT	Somatic cell nuclear transfer
ZNF	Zinc finger nucleases
